# Resuming cadaver dissection during a pandemic

**DOI:** 10.1080/10872981.2020.1842661

**Published:** 2020-10-27

**Authors:** Georgina Bond, Thomas Franchi

**Affiliations:** a Human Anatomy Technician, Medical Teaching Unit, Academic Unit of Medical Education, The University of Sheffield, Sheffield, UK; b Medical Student, The Medical School, The University of Sheffield, Sheffield, UK; c Human Anatomy Demonstrator, Department of Biomedical Science, The University of Sheffield, Sheffield, UK

**Keywords:** cadaver dissection, anatomical education, medical students, covid-19, pandemic

## Abstract

Methods of anatomical education have, as with many facets of normal life, been forced to evolve rapidly due to the Covid-19 pandemic. Whilst some authors claim that cadaver dissection is now under threat, we believe the centuries-old practice can and must be upheld.

## Letter

We read with hesitation the letter shared by Ooi and Ooi [1], outlining their perspectives on the future of anatomy teaching through cadaveric dissection as a result of the Covid-19 pandemic. Notably, we do not share the same cynical outlook regarding medical schools’ ability to adapt to the situation and disagree with a number of the assertions made. As a Human Anatomy Technician (GB), and Medical Student and Human Anatomy Demonstrator (TF), we have experienced first-hand how Covid-19 has affected medical education [2], and are currently working in-line with the ever-changing laws and restrictions which are being implemented in the UK.

Although the UK went into lockdown on 23 March 2020, The University of Sheffield ceased face-to-face teaching a week prior. This decision was implemented swiftly, meaning all dissection classes were abruptly cancelled for the foreseeable future. This cancellation came into effect whilst the medical students, amongst other student cohorts who undertake cadaveric dissection, still had over 8 weeks of practical teaching to complete. The lockdown had many further implications, as Ooi and Ooi outline [1], including that body donation programmes were closed. However, in their letter, it was incorrectly stated that the Human Tissue Authority (HTA) contacted medical schools to inform them that they were not allowed to accept donations. In reality, institutions were unable to accept donations as staff were furloughed or forced to work from home, as per government-issued guidance. The HTA-issued guidance regarding body donations was sent to institutions on 2 April 2020 and stated that establishments had to consider how they would manage enquiries [3]. It advised that information should be disseminated to families of potential donors to explain the current situation and we, at The University of Sheffield, did this via out-of-office email replies and voicemails on staff office phones.

The major concern arising from the cessation of accepting body donors, was that institutions may be unable to meet the donor body requirements to accommodate all courses. Whilst Ooi and Ooi rightly stated that this would affect the delivery of teaching in the future [1], they wrongly and worryingly suggested that this has led to cadaveric dissection classes being cancelled. Although the Covid-19 pandemic is ongoing, most universities have opted to reopen this academic year, albeit with limited face-to-face teaching. Many institutions are giving priority to practical and clinical classes such as cadaveric dissection and anatomy laboratory sessions. Indeed, other authors have highlighted that not only is cadaver dissection during Covid-19 possible, but it is also essential [4]. Despite suboptimal numbers of donor bodies as compared to pre-Covid-19 cadaver-to-student ratios, this certainly has not meant that dissection has been cancelled. Social distancing requirements have severely reduced class sizes and cohorts have been split into multiple time slots throughout the week to ensure all students still gain this valuable experience. The practice of cadaver dissection is therefore alive and well.

As the pandemic continues, only some UK universities have restarted their body donation programmes, potentially due to the uncertainty surrounding the potential of renewed lockdowns or due to concerns around the ability to guarantee that donor bodies are negative for Covid-19. This causes an ongoing issue, as for those institutions who usually accept large numbers of donors, the longer body donation programmes remain closed, the longer it will take to recover. This therefore creates potential for Covid-19 to affect not only this academic year, but also cause significant problems for subsequent years. Nevertheless, this does not mean that dissection has or will be cancelled completely as Ooi and Ooi allude to [1]. The authors do however make very valid points about the benefits of dissection and the advantages it affords to students, which we unreservedly agree with. Students gain not only anatomical knowledge, but also life lessons in ethics and humanity [2,5]. In light of this, despite the pandemic having and continuing to cause problems with the delivery of medical education, institutions and educators are continuing to seek ways to ensure pedagogies such as dissection are integrated into the modified curriculum.

Moving forwards, universities will undoubtedly rely heavily on technologies such as online dissection videos and anatomical lectures. However, dissection is too rich in educational opportunity and benefit for institutions to indefinitely curtail its use as an anatomical pedagogy [2,4,5].

‘*We owe it to our medical students, to their future patients, and to our communities to continue providing dissection-centered anatomy education*’ [4].
